# *Staphylococcus aureus* and *Staphylococcus lugdunensis* Act in Concert to Disrupt the Nasal Epithelial Barrier

**DOI:** 10.1002/clt2.70177

**Published:** 2026-05-22

**Authors:** Sintayehu Ambachew, Mahnaz Ramezanpour, Clare M. Cooksley, Gohar Shaghayegh, Isabella Burdon, Emma F. Barry, Kevin Aaron Fenix, P. J. Wormald, Alkis J. Psaltis, Sarah Vreugde

**Affiliations:** ^1^ Adelaide Medical School Faculty of Health and Medical Sciences The University of Adelaide Adelaide Australia; ^2^ The Department of Surgery—Otolaryngology‐Head and Neck Surgery University of Adelaide and the Basil Hetzel Institute for Translational Health Research Central Adelaide Local Health Network Adelaide South Australia Australia

**Keywords:** biofilms, Chronic rhinosinusitis, exoproteins, Nasal epithelial cells, *Staphylococcus species*

## Abstract

**Introduction:**

Chronic rhinosinusitis (CRS) pathophysiology and its link to microbiome is an area of ongoing investigation. Certain pathogens, in particular *Staphylococcus aureus* described to contribute to recalcitrant CRS. In addition, different species of coagulase negative *staphylococci* (CoNS) are frequently isolated from the sinonasal cavity of CRS patients. However, the influence of *Staphylococcal species* coexisting in the same niche on the inflammatory process remains unclear. The aim of this study was to explore the impact of exoproteins from various *Staphylococcus* species isolated from the same patients on the mucosal barrier.

**Methods:**

*Staphylococcal* species isolated from CRS and control patients were cultured from sinus swabs in planktonic and biofilm forms, and their exoproteins extracted. Primary human nasal epithelial cells (HNECs) from CRS patients were cultured at an air‐liquid interface (ALI) and exposed to 20 μg/mL exoproteins or control. Barrier disruption and cytotoxicity were assessed by measuring the transepithelial electrical resistance (TEER), passage of fluorescein labeled dextrans and lactate dehydrogenase (LDH) levels. IL‐ 6 concentration was measured employing ELISA. Patient's matched sinonasal tissue samples were analyzed with flow cytometry to detect and quantify immune cells.

**Results:**

Forty‐four Staphylococcal species were isolated from 22 CRS and control patients including: 22 *S. aureus*, 12 *S. epidermidis*, and 10 *S. lugdunensis*. 15 out of 22 *S. aureus* exoproteins significantly enhanced cytotoxicity, reduced TEER values and increased paracellular permeability compared to control (*p* < 0.05). By contrast, *S. epidermidis* and *S. lugdunensis* exoproteins caused either mild or negligible effects on the TEER values, cell viability, and paracellular permeability. However, *S. lugdunensis* exoproteins induced significantly higher IL‐6 compared to control. Correlation analysis indicated *S. aureus and S. lugdunensis* from the same patient acted in concert to disrupt the nasal epithelial barrier and induce toxicity.

**Conclusion:**

This study shows the significant and detrimental impact of the presence of *S. aureus* exoproteins on nasal epithelial cell barrier function. *S. aureus* and *S. lugdunensis* isolated from the same patients acted in concert to affect the nasal barrier and inducing toxicity.

**Abbreviations**
ALI: air liquid interface
CoNS: coagulase negative staphylococci
CRS: chronic rhinosinusitis
CRSsNP: chronic rhinosinusitis without polyps
CRSwNP: chronic rhinosinusitis with polyps
CTRL: Control patients
ELISA: Enzyme linked immunosorbent assay
FITC‐ dextran: Fluorescein isothiocyanate‐Dextran
HGT: Horizontal gene transmission
HNECs: Human nasal epithelial cells
IL‐6: interleukin‐ 6 levels
LDH: Lactate dehydrogenase assay
MALDI‐TOF MS: matrix‐assisted laser desorption ionization‐time of flight mass spectrometry
OD: optical density
SNOT‐22: Sino – Nasal Outcome Test
TEER: transepithelial electrical resistance
TJs: tight junctions
TSA: tryptic soy agar
TSB: tryptic soy broth

## 1 Introduction

Chronic rhinosinusitis (CRS) is a common disorder impacting the mucosal lining of the paranasal sinuses [1, 2] and is classified as CRS with nasal polyps (CRSwNP) and CRS without nasal polyps (CRSsNP), frequently associated with Th2‐ and Th1‐mediated inflammatory responses, respectively [3].

The interplay between innate and adaptive immunity plays a major role in the pathogenesis of CRS [4]. Mucosal barriers form an integral part of the innate immune system and protect host tissues from exogenous insults [5]. Maintaining the health and cellular integrity of these barriers is core to this function of the innate immune system. In the context of CRS, infection with pathogenic bacteria can compromise the epithelial barrier, perpetuating and dysregulating the inflammatory response [6, 7].

Numerous studies have reported epithelial barrier disruption as the principal pathophysiological feature of CRS [8, 9, 10]. It is often accompanied by alterations in the nasal microbiome and its composition [10, 11]. However, the interplay between the microbiome and epithelial barrier function in the aetiopathogenesis of CRS remains unclear. The bacterial species colonizing the sinonasal epithelium may vary due to several factors such as individual health status, geographical location, and environmental conditions. Nonetheless, bacterial genera such as *Staphylococcus, Corynebacterium*, *Streptococcus,* and *Propionibacterium,* along with species like *Haemophilus influenzae and Moraxella catarrhalis*, have been identified as predominant bacteria in CRS.

Staphylococci are often considered as nasal commensals, but some strains can become opportunistic pathogens, causing infections and disrupting sinonasal microbial balance [12, 13]. *Coagulase negative Staphylococcus (CoNS)* are frequently isolated from the sinonasal cavities of CRS patients [14, 15]. While individual susceptibility to bacterial infection varies [16], the contribution of different *Staphylococcus* species concurrently colonizing the same sinonasal epithelium in driving the inflammation remains unclear.

Within a shared ecosystem, various *Staphylococcal* species compete for nutrients and resources in order to survive in the same niche, impacting their growth rate and abundance [17]. Certain *Staphylococci* produce substances to inhibit the growth or virulence of other antagonizing species. For instance, *S. lugdunensis* has been demonstrated having antagonistic effects though reported to exhibit virulence similar to *S. aureus* [18]. An in vitro study has shown that Lugdunin from *S. lugdunensis* inhibits *S. aureus* growth. Similarly, the antimicrobial peptide nukacin IVK45, produced by *S. epidermidis*, was found to possess inhibitory activity against *S. aureus* growth [19, 20].

On the other hand, synergism between *Staphylococcal* species has also been identified whereby their combined virulence can increase the severity of disease beyond the impact of colonization by any individual species alone [21, 22, 23]. This synergy is largely due to biofilm formation and horizontal gene transfer (HGT) [24]. Analysis of the genomes of 324 *S. aureus* and *S. epidermidis* isolates uncovered extensive exchange of mobile genetic elements between the species, including sharing of nearly half of their genetic makeup [25]. These situations contribute to the complex nature of the microbial community, impacting the differential or cumulative pathogenic potential of various *Staphylococci* and challenging therapeutic options [26].

*S. aureus* colonizes up to 64% of CRSwNP patients [27] and contributes to refractory disease through exotoxins like *α*‐, *β*‐, and *γ*‐toxin. These damage epithelial barriers, disrupt ion balance, and induce inflammation [21, 22, 28]. By impairing ciliary function and triggering immune evasion, they play a central role in CRS pathogenesis [21, 22]. Moreover, recent studies have demonstrated strain‐specific effects of *S. epidermidis* and *S. lugdunensis* on mucosal barrier integrity [23], despite their classification as commensal organisms. Similarly, *S. aureus* can colonize the sinuses without causing inflammation [13]. Our previous research shows that not all *S. aureus* strains are the same, and that strain‐dependent variability in biofilm formation is associated with differences in inflammatory responses [29].

However, the coexistence of *S. aureus* and other CoNS family members in the same sinonasal niche and how it contributes to CRS pathogenesis remains unknown. This study aims to determine whether CoNS strains differ in pathogenicity and how this relates to the pathogenic potential of *S. aureus*.

## 2 Materials and Methods

### 2.1 Ethical Statement and Data Collection

Ethics approval for this study was obtained from the Central Adelaide Local Health Network Human Research Ethics Committee (CALHN HREC) (Ref no. 13604) and Calvary HREC (Ref no. 19‐CHREC‐E003). All isolates and cells were collected from patients after obtaining written informed consent. The diagnostic criteria for CRSwNP, and CRSsNP were defined by the American Academy of Otolaryngology and Head and Neck Surgery and the European Position Statement (EPOS) on CRS [30, 31]. Individuals having upper respiratory conditions other than CRS, such as septal deviation, or those undergoing skull base surgery for pituitary tumors, were used as control subjects. Disease severity was measured using the 22‐item Sino‐Nasal Outcome Test (SNOT‐22) questionnaire [32], Lund–Mackay (LM) [33], and Lund–Kennedy (LK) [34, 35] where available. We categorized SNOT‐22 scores as mild (< 50), and severe (> 50) [36].

### 2.2 Bacterial Strains and Growth Conditions

*Staphylococcus* specie*s* including *S. aureus, S. epidermidis* and *S. lugdunensis* isolated from the sinonasal niche of patients with CRSwNP, CRSsNP and control subjects were used in this study (Table 1). Bacterial strains were cultured on tryptone soy agar (TSA) and identified by matrix‐assisted laser desorption ionization‐time of flight mass spectrometry (MALDI‐TOF MS) [37]. Following overnight incubation at 37°C with 5% CO_2_, single colonies were isolated and stored at −80°C in a 1 mL aliquot of freeze media (TSB with 20% glycerol).

**TABLE 1:** Patient demographics and clinical characteristics.

Characteristics	CRSsNP, *N* (%)	CRSwNP, *N* (%)	CTRL, *N* (%)	Total, *N* (%)
Number of subjects	7 (31.8)	10 (45.5)	5 (22.7)	22 (100)
Sex (F/M)	2/5	5/5	1/4	8/14
Asthmatic	3 (13.6)	6 (27.3)	1 (4.5)	10 (45.5)
Allergic	3 (13.6)	3 (13.6)	0 (0)	6 (27.3)
Aspirin sensitivity	1(4.5)	1 (4.5)	0 (0)	2 (9)
GORD	1 (4.5)	3 (13.6)	1 (4.5)	5 (22.7)
Smoking (past smoker)	4 (18.2)	5 (22.7)	3 (13.6)	12 (54.5)
S.A only	0 (0)	5 (22.7)	0 (0)	5 (22.7)
S.A + S.E	3 (13.6)	2 (9.1)	2 (9.1)	7 (31.8)
S.A + S.L	2 (9.1)	1 (4.5)	2 (9.1)	5 (22.7)
S.A + S.E + S.L	2 (9.1)	2 (9.1)	1 (4.5)	5 (22.7)
Total S.A	7	10	5	22
Total S.E	5	4	3	12
Total S.L	4	3	3	10

Abbreviations: CRSsNP: chronic rhinosinusitis without polyps, CRSwNP: chronic rhinosinusitis with polyps, CTRL: Control patients, GORD: Gastro‐esophageal reflux disease, S.A: *S. aureus*, S.E*: S. epidermidis*, S.L*: S. lugdunensis*.

### 2.3 Growth of Bacteria in Planktonic and Biofilm Forms

Bacteria were cultured on TSA from frozen stock and incubated at 37°C for 18–24 h. For the preparation of planktonic bacteria, individual colonies were inoculated into a 50 mL tube containing 10 mL of sterile broth medium. The tubes were then incubated overnight at 37°C with agitation (180 rpm), followed by optical density readings using a *smartSpec* ^*tm*^ *3000* spectrophotometer (Bio‐Rad Laboratories, Hercules, CA, USA) at 600 nm. Growth curves were then constructed for each strain. Subsequently, extraction of exoprotein from bacteria was carried out during the stationary phase of bacterial growth.

For the biofilm forms, a suspension of each isolate was prepared using 1.0 ± 0.1 McFarland Units using McFarland densitometer (Bio‐Strategy, Auckland, New Zealand), diluting it in 1:15 ratio in TSB, then 2 mL was transferred into each of 3 wells in a 6 ‐well plate (Corning Incorporated, Corning, NY, USA). The bacteria were incubated on a rotating plate set at 70 rpm for 48 h at 37°C using Orbital Mixer Incubator (Adelab Scientific, Thebarton, SA, Australia). After confirming biofilm formation, exoproteins were collected.

### 2.4 Preparation of *Staphylococcus* Exoproteins and Protein Assay

The supernatant containing exoproteins from biofilms and planktonic cells was collected by centrifugation (4000 × g, 4°C for 10 min) and filtration with a 0.2 μm pore size Millex polyethersulfone syringe filters (Merck Millipore, Burlington, MA, USA). This supernatant was then passed through a pre‐rinsed 3‐kDa Ultra Filters Vivaspin concentrator (Thermo Fisher Scientific, Rockford, IL, USA) by centrifugation at 4000 × g, 4°C for 1–2 h to collect exoproteins. Final volume of 1 mL and 500 μL of exoprotein/supernatant in planktonic and biofilm forms, respectively, was achieved from each sample. Then, the protein content was determined using NanoOrange Protein Quantitation Kit (Thermo Fisher Scientific, Waltham, MA, USA), according to manufacturer's instructions. The extracted proteins were stored as single‐use aliquots at −80°C until use. All reactions were carried out in duplicate.

### 2.5 Human Nasal Epithelial Cell Culture

HNEC cultures were established as previously described [38]. Briefly, cells were harvested from nasal mucosa by gentle brushing (CRSwNP patients). The nasal brushings were then suspended in Nasal Epithelial Growth Media (STEMCELL Technologies Australia Pty. Ltd, Tullamarine, VIC, Australia). Subsequently, the extracted cells underwent monocyte depletion by incubating them with culture dishes coated with anti‐CD68 antibodies (Dako, Glostrup, Denmark) at 37°C for 20 min. HNECs were then cultivated with Pneuma‐ Cult‐ Ex Plus medium in collagen coated T75 cell culture flasks (Corning Incorporated, Corning, NY, USA) at 37°C with 5% CO_2_ until reaching confluence. The medium also included PneumaCult‐Ex Plus 50x Supplement, antifungal (amphotericin B), and antibiotics (penicillin, and streptomycin) (Thermo Fisher Scientific, Scoresby, VIC, Australia).

### 2.6 Air Liquid Interface Culture

Air Liquid Interface (ALI) cultures were established as previously described [38]. Briefly, 9.5 × 10^4^ HNECs were seeded in 100 μL PneumaCult Ex Plus medium into the apical chamber of Transwell plates (6.5 mm insert, Corning Incorporated), with 500 μL of Ex Plus medium added to the basal chamber. The plates were incubated for about 3 days at 37°C with 5% CO_2_. Subsequently, the apical medium was removed and 500 μL PneumaCult‐ALI differentiation medium was added to the basal chamber and replaced every second day. HNECs at ALI were maintained for about 17–21 days for development of tight junctions. Only wells with baseline resistance measurements greater than 700 *ω*/cm^2^ were used to conduct experiments.

### 2.7 Trans‐Epithelial Electrical Resistance (TEER)

TEER was measured by using an EVOM volt‐ohmmeter (World Precision Instruments, Sarasota, FL, USA). A 100 μL volume of Pneumacult medium was supplied to the apical chamber of ALI cultures to establish an electrical circuit across the cell monolayer and into the basal chamber. Pneumacult EX‐Plus medium and 2% Triton X‐100 were used for the negative and positive controls, respectively. We first conducted a pilot study, applying 20, 40 and 60 μg/mL exoproteins (or control) to HNEC cultures to identify a dose that induced consistent measurable effects on TEER for at least some strains without being overly toxic (as measured in LDH assays). After optimization, 20 μg/mL exoproteins were added to ALI cultures, followed by TEER measurements at different time intervals from time 0 to 3 h, with the cultures maintained at 37°C throughout the measurement period. The TEER measurements were conducted in triplicate (3 biological repeats with HNECs from 3 independent donors).

### 2.8 Dextran‐Fluorescein Isothiocyanate (FITC) Permeability Assay

Paracellular permeability was examined by determining the apical‐to‐basolateral flux of FITC‐ dextran 4 kDa (Merck Life Science, Bayswater, VIC, Australia) as previously described [39]. In brief, after treating the cells with exoproteins for 3 h, the upper chambers were loaded with 3 mg/mL of FITC‐dextran and incubated for 2 h at 37°C. 40 μL from each sample was retracted from the bottom chamber and transferred in duplicate to a 96‐well plate (Greiner Bio‐One, Kremsmünster, Austria). Finally, a microplate fluorometer FLUOstar OPTIMA (BMG LABTECH, Ortenberg, Germany), was used to measure the fluorescence. The experiment was carried out in triplicate.

### 2.9 Cell Cytotoxicity Assay

The medium in the basal chambers was collected following the last TEER measurements (3 h) and was subjected to lactate dehydrogenase (LDH) release assay using LDH assay kit from Promega Corporation (Madison, WI, USA) according to the manufacturer's instructions. Relative viability of cells was calculated by comparing the LDH levels of exoprotein‐treated samples with those of negative controls. The experiment was conducted in triplicate.

### 2.10 Measurement of Cytokine Interleukin‐6 Levels

Enzyme‐linked immunosorbent assay (ELISA) was employed in order to quantify the amount of interleukin‐6 (IL‐6) after treating HNECs with exoproteins obtained from both planktonic and biofilm bacteria conducted according to manufacturer's instructions (Thermofisher Scientific, Waltham, MA, USA). The experiment was repeated three times.

### 2.11 Flow Cytometry Analysis

Flow cytometry (FACS) data from our previous study were utilized for this study [37]. Briefly, fresh nasal polyps or mucosal samples from CRS and control patients, respectively, were processed into single cell suspensions followed by flow cytometry staining against various immune cell types using protocols detailed in [29, 40]. Samples were acquired using a BD FACS Canto II (BD Bioscience, San Jose, CA, USA). Flow cytometry data were analyzed using FlowJo v10 (BD Bioscience, San Jose, CA, USA).

### 2.12 Data Analysis

GraphPad Prism (GraphPad Software, San Diego, CA, USA) was used to analyze the data. Descriptive statistics for frequency data were calculated to summarize the dataset. A one‐way/two‐way ANOVA was conducted to evaluate differences among the comparison groups. Multiple comparisons were performed using the Dunnett test to compare the impact of each *Staphylococcus* exoprotein with a control or/and the Tukey test to compare all pairs of groups. The findings were reported as mean difference ± standard error (MD ± SE). Correlation analysis, ordinal logistic regression, and mixed linear effect models were used to assess the association between dependent and independent variables using GraphPad Prism, and the stats models package in Python version 3.3.1. In addition, principal component analysis was conducted employing R software (version 2024.12.0).

## 3 Results

### 3.1 Demographic and Clinical Characteristics of the Patients

Clinical isolates were obtained from 22 CRS patients (CRSwNP, CRSsNP) and controls. The mean participant age and SNOT‐22 score were 49.36 ± 21.28 years and 36.68 ± 24.22, respectively. From these patients a total of 44 *Staphylococcus* species were isolated including 22 *S. aureus*, 12 *S. epidermidis* and 10 *S. lugdunensis*. Five patients were infected with *S. aureus* only, 7 patients were infected with *S. aureus* and *S. epidermidis*, 5 with *S*. *aureus* and *S. lugdunensis,* and 5 patients were infected with all three species. Patient demographics and clinical characteristics are summarized in Table 1.

### 3.2 Effects of Exoproteins on Nasal Epithelial Barrier Function

#### 3.2.1 *S. aureus* Exoproteins had the Strongest Effect in Reducing TEER Values, Which Is Predictive of Worse Disease Severity

We first aimed to compare the effects of exoproteins harvested from *S. aureus, S. epidermidis,* and *S. lugdunensis*, grown in planktonic or biofilm forms, on epithelial barrier integrity.

**FIGURE 1:**
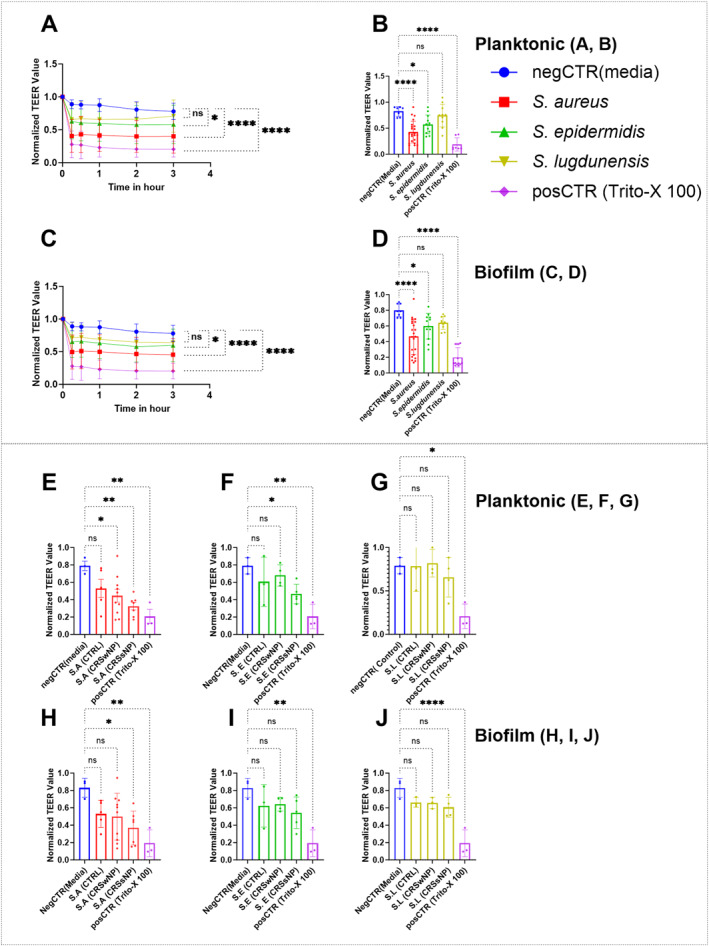
Transepithelial electrical resistance (TEER) of human nasal epithelial cell at air‒liquid interface (HNEC‐ALI) cultures following exposure to *Staphylococcus* species exoproteins. TEER values over 3 h (A, C) and at the 3‐h time point (B, D) following application of exoproteins from planktonic (A, B) and biofilm (C, D) forms of *Staphylococcus* species were analyzed using one/two‐way ANOVA followed by Tukey's multiple comparisons test. Results represented as mean ± SEM. *p ≤ 0.05, ****p ≤ 0.0001; ns: not statistically significant. In planktonic (E, F, and G) and *biofilm* (H, I, and J) forms show the effects of exoproteins from different *Staphylococcus species*, isolated from patients with varying CRS status and controls, on HNEC‐ALI cultures. TEER values were normalized to pre‐treatment levels (*t* = 0) to account for baseline variation. negCTR: untreated cells in Ex Plus medium; posCTR: cells treated with 1% Triton X‐100. HNEC‐ALI cultures were obtained from three independent donors (*n* = 3). These were analyzed using Dunnett's multiple comparisons test (*p ≤ 0.05, **p ≤ 0.01, ****p ≤ 0.0001; ns: not statistically significant). CRSsNP: CRS without nasal polyps, CRSwNP: CRS with nasal polyps, CTRL: control patients, S.A: *S. aureus*, SE: *S. epidermidis*, SL: *S. lugdunensis*.

Using planktonic derived exoproteins, *S. aureus* exoproteins caused a significant reduction in average TEER values within 15 min compared to the negative control, an effect that persisted for 3 h (MD ± SE of 0.38 ± 0.046; *p* < 0.0001). A significant reduction was also observed for *S. epidermidis* exoproteins (0.199 ± 0.059, *p* = 0.0124 at 3 h). In contrast, *S. lugdunensis* exoproteins did not significantly alter TEER values (Figure 1A).

Similarly, biofilm‐derived exoproteins from *S. aureus* led to a significant reduction in average TEER values within 15 min compared to control, which also persisted for 3 h (0.326 ± 0.045, *p* < 0.0001). *S. epidermidis* biofilm exoproteins also reduced TEER values (0.18 ± 0.05; *p* = 0.0104) at 3 h, while *S. lugdunensis* biofilm exoproteins showed no significant effect (Figure 1C).

For each of the species tested, there was strain‐dependent variability in the effect on TEER values at the 3‐h time point in both planktonic and biofilm forms (Figure 1B and D). Analysis showed that the average effect on TEER values of the various species was not influenced by whether those species were isolated in the context of mono‐infections or polymicrobial infections involving more than one *Staphylococcus* species (Supporting Information S1: Figure S1 and Supporting Information S2: Table S1).

We then evaluated how disease status influenced the effect of exoproteins from various *Staphylococcus* species on the mucosal barrier. Exoproteins from *S. aureus* isolated from CRSsNP and CRSwNP patients in planktonic forms significantly reduced the TEER value compared to negative control (0.46 ± 0.13; *p* = 0.0018, and 0.34 ± 0.128; *p* = 0.0118) respectively. In contrast, exoproteins from *S. aureus* harvested from control patients did not significantly reduce TEER values (0.26 ± 0.139, *p* = 0.0748) (Figure 1E). Exoproteins from *S. epidermidis* in planktonic forms from CRSwNP and control patients did not significantly reduce TEER compared to negative control (0.11 ± 0.119, *p* = 0.7492 and 0.182 ± 0.127, *p* = 0.4266 respectively). However, those from CRSsNP patients showed a reduction in TEER compared to control (0.323 ± 0.113, *p* = 0.0422; Figure 1E–G). In biofilm forms, exoproteins from *S. aureus* harvested from CRSsNP patients showed a significant reduction compared to experimental control (0.46 ± 0.147, *p* = 0.0144). However, exoproteins from *S. epidermidis* and *S. lugdunensis* biofilms isolated from either CRSsNP, CRSwNP or control patients did not show a significant reduction in TEER values compared to control (*p* > 0.05; Figure 1H–J).

We then performed ordinal logistic regression analysis to evaluate the relationship between barrier dysfunction represented by TEER values and the presence of disease. Lower TEER values were associated with bacteria that were isolated from CRS patients (OR = 0.479, *p* = 0.049). Among the various species, *S. aureus* exoproteins from planktonic forms were the main drivers of this statistic with this subset of the data being more significantly associated with lower TEER values (OR = 0.287, *p* = 0.003). Furthermore, lower TEER values were associated with greater SNOT‐22 disease severity scores (OR = 0.508, *p* = 0.024); that is, there is 49% reduction in the TEER values of patients with severe SNOT‐22 score category as compared to the mild category (Table 2).

**TABLE 2:** Significant relationships identified by ordinal logistic regression.

Data set	Outcome	OR	SE	95% LCL	95% UCL	*p*‐value
All bacteria	CRS binary	0.479	1.45	0.231	0.996	0.049
All bacteria	SNOT22 severity	0.508	1.35	0.282	0.916	0.024
S.A planktonic form	SNOT22 severity	0.287	1.52	0.126	0.655	0.003

*Note:* All bacteria: *S. aureus, S. epidermidis and S. lugdunensis*.

Abbreviations: LCL: lower confidence limit, S.A: *S. aureus*, SE: Standard error, UCL: upper confidence limit.

Further, a linear mixed effect model indicated that isolates harvested from CRSsNP patients were predictive of lower TEER values than CRSwNP and controls with a *p* = 0.01 and *p* = 0.003, respectively (Table 3).

**TABLE 3:** TEER as an outcome having a significant relationship with predictors identified by linear mixed effect model.

Predictor	Β	SE	95% LCL	95% UCL	*p*‐value
[CRSsNP versus] CRSwNP	1.26	1.09	1.06	1.50	0.01
[CRSsNP versus] CTRL	1.34	1.11	1.10	1.63	0.003

Abbreviations: CRSsNP: chronic rhinosinusitis without polyps, CRSwNP: chronic rhinosinusitis with polyps, CTRL: Control patients, LCL: Lower confidence limit, UCL: upper confidence limit.

#### 3.2.2 *S. aureus* and *S. lugdunensis* Exoprotein‐Mediated Effect on TEER Values are Correlated When Isolates are Harvested From the Same Patient

**FIGURE 2:**
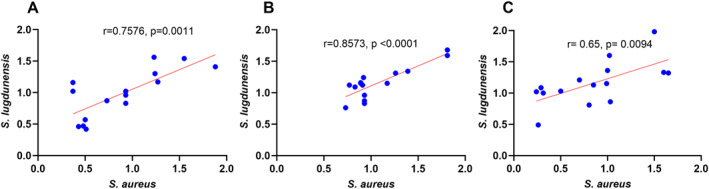
Linear correlations between the TEER values of HNEC‐ALI cultures exposed to exoproteins from *S. aureus* and *S. lugdunensis* during co‐infections. Correlations were assessed for dual‐species infections (A and B) in planktonic and biofilm forms, as well as for triple‐species biofilms (C). Pearson's correlation coefficient was used for the analysis.

Given the observed variability among the various strains in their effect on TEER values, we then wanted to evaluate whether different *Staphylococcal* species harvested from the same patient had similar effects on TEER values. A strong positive correlation was observed between the TEER values of HNECs exposed to exoproteins of *S. aureus* and *S. lugdunensis* in both planktonic and biofilm forms of isolates from the same patient (*r* = 0.7576, *p* = 0.0011, and *r* = 0.8573, *p* < 0.0001, respectively) (Figure 2A–B).

Similarly, in patients that harbored all 3 *Staphylococcus* species, we observed a moderate positive correlation between the TEER values of HNECs exposed to exoproteins from *S. aureus* and *S. lugdunensis* in biofilm form (*r* = 0.6448, *p* = 0.0094) (Figure 2C). In contrast, no correlations were observed between the TEER values of HNECs exposed to exoproteins from *S. aureus* and *S. epidermidis* in either planktonic or biofilm form, nor between *S. epidermidis* and *S. lugdunensis* (Supporting Information S1: Figure S2).

### 3.3 *S. aureus* and *S. lugdunensis* Exoproteins Co‐Isolated From the Same Niche are Correlated to Enhance Paracellular Permeability

**FIGURE 3:**
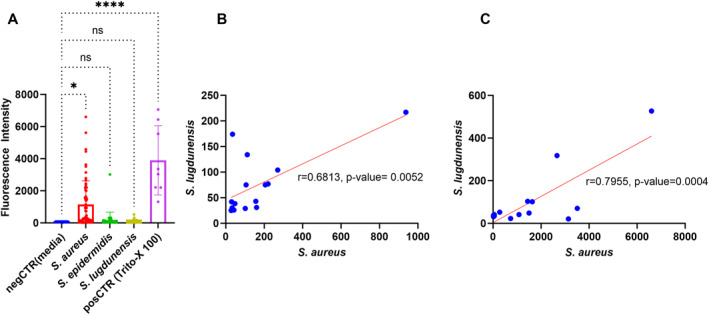
Paracellular permeability of human nasal epithelial cell at air‒liquid interface (HNEC‐ALI) cultures following *Staphylococcus* species exoprotein exposure. Paracellular permeability was measured by monitoring the passage of fluorescein isothiocyanate (FITC)‐dextran across epithelial barrier. The figure illustrates the impact of *Staphylococcus* species exoproteins on HNEC‐ALI cultures derived from planktonic forms (A). Ex Plus medium was used as a negative control, and 1% Triton X‐100 served as a positive control. HNEC‐ALI cultures were obtained from three independent donors (*n* = 3). (B and C) show the correlation analysis of paracellular permeability in HNEC‐ALI cultures exposed to *S. aureus* and *S. lugdunensis* during dual‐species (B) and triple‐species (C) infections in planktonic form. Pearson's correlation coefficient was used for the correlation analysis. Data were analyzed using one‐way ANOVA followed by Dunnett's multiple comparisons test (*p ≤ 0.05, ****p ≤ 0.0001; ns: not statistically significant).

Next, we determined the effect of *Staphylococci* exoproteins on the paracellular permeability. *S. aureus* exoproteins in planktonic form caused significant elevation in paracellular permeability as compared to negative control (−1103 ± 421.2, *p* = 0.0130). However, *S. epidermidis* and *S. lugdunensis* exoproteins from planktonic forms did not enhance the paracellular permeability compared to control (−133.3 ± 467.4, and −40.95 ± 485.8 respectively, *p* > 0.05; Figure 3A). Paracellular permeability after exposure to exoproteins from various species were comparable whether the exoproteins were derived from mono‐infections or from dual or triple infections (Supporting Information S1: Figure S3).

A strong positive correlation was observed between the paracellular permeability of HNECs exposed to exoproteins of *S. aureus* and *S. lugdunensis* from isolates that were harvested from the same patient in dual infections (*r* = 0.6813, *p* = 0.0052) and in triple infections (*r* = 0.7955, *p* = 0.0004) in planktonic forms (Figure 3B–C).

No correlation was observed between the paracellular permeability of HNECs exposed to exoproteins of *S. aureus* and *S. epidermidis* nor to *S. epidermidis* and *S. lugdunensis* in the context of dual and triple infections in both biofilm and planktonic forms (Supporting Information S1: Figure S4).

### 3.4 *S. aureus* and *S. lugdunensis* Exoproteins Co‐Isolated From the Same Niche are Correlated to Enhanced Cellular Toxicity

Exoproteins harvested from *S. aureus* in planktonic forms and all 3 species in biofilm forms induced a significant cytotoxic effect compared to negative control (37.26 ± 10.7, *p* = 0.0093 for planktonic *S. aureus*, and 30.54 ± 6.65, 22.02 ± 6.976 and 24.38 ± 7.114, *p* = 0.0003, 0.0226, and 0.0110 for *S. aureus, S. epidermidis* and *S. lugdunensis* biofilm exoproteins respectively). Viability of cells was similar after being challenged with exoproteins from the various *species* according to whether they were harvested alone or together with other *species* in the niche in dual or triple infections (Supporting Information S1: Figure S5).

**FIGURE 4:**
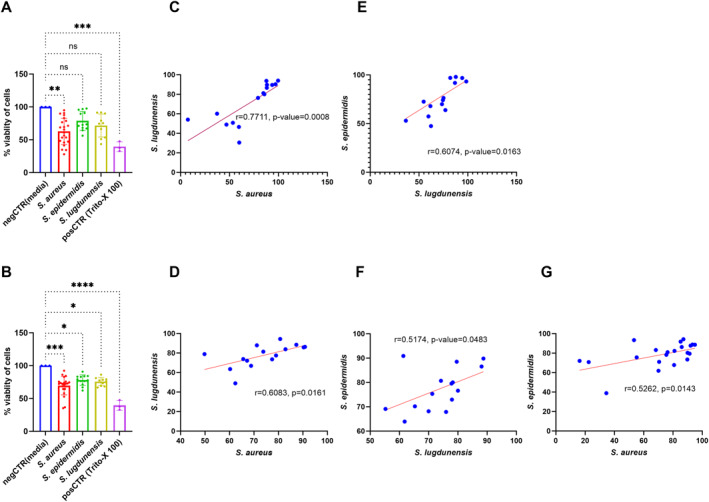
Cell viability of human nasal epithelial cell at air‒liquid interface (HNEC‐ALI) cultures following exposure to exoproteins of *Staphylococci* species in planktonic (A) and biofilm (B) forms. The lactate dehydrogenase (LDH) assay was used to determine cell viability. Cell viability (%) was estimated relative to cells maintained in culture medium (negative control). The correlation analysis describes cell viability in HNEC‐ALI cultures exposed to *S. aureus* and *S. lugdunensis* in both planktonic (C) and biofilm (D) forms during dual infections. In the context of triple infections, exposure to *S. lugdunensis* and *S. epidermidis* in planktonic (E) and biofilm (F) forms, and *S. aureus* and *S. epidermidis* in biofilm (G) forms was also assessed. Ex Plus medium was used as the negative control, and 1% Triton X‐100 served as the positive control. HNEC‐ALI cultures were obtained from three independent donors (*n* = 3). Pearson's correlation coefficient was used for the correlation analysis. Data were analyzed using one‐way ANOVA followed by Tukey's multiple comparisons test (*p ≤ 0.05, **p ≤ 0.01, ***p ≤ 0.001, ****p ≤ 0.0001; ns: not statistically significant).

We observed a moderate positive correlation between the percentage viability of cells exposed to *S. aureus* and *S. lugdunensis* exoproteins in the context of dual infection (*r* = 0.7711, *p* = 0.0008, and *r* = 0.6083, *p* = 0.0161, for planktonic and biofilm forms respectively). A moderate positive correlation was also observed between the percentage viability of cells exposed to *S. aureus* and *S. epidermidis* exoproteins in biofilm form during dual infection (*r* = 0.5262, *p* = 0.014) and between *S. epidermidis* and *S. lugdunensis* in the context of triple infections (*r* = 0.6074, *p* = 0.0163, and *r* = 0.5174, *p* = 0.0483, for planktonic and biofilm forms, respectively; Figure 4). No significant correlations were found between the percentage viability of cells exposed to exoproteins of *S. aureus* and *S. epidermidis* during dual infections in planktonic form. Similarly, during triple infections, no significant correlations were observed between *S. aureus* and *S. epidermidis* in planktonic form, nor between *S. aureus* and *S. lugdunensis* in both planktonic and biofilm forms (Supporting Information S1: Figure S6).

### 3.5 *S. aureus* and *S. lugdunensis* Co‐Isolated From the Same Niche Correlated With Induction of IL‐6

**FIGURE 5:**
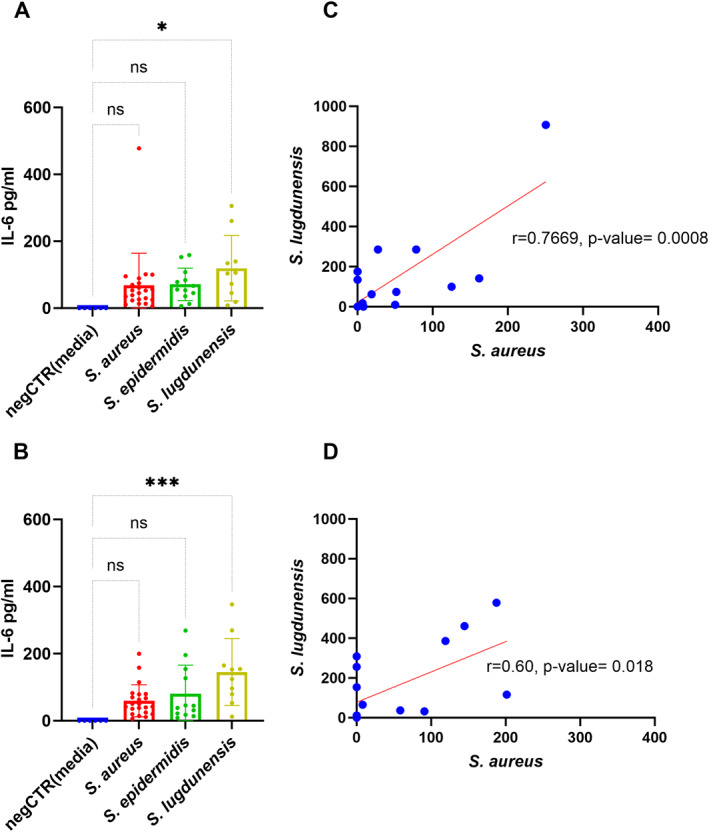
Interleukin‐6 (IL‐6) release in human nasal epithelial cell air–liquid interface (HNEC‐ALI) cultures mediated by *Staphylococcus* species exoproteins. IL‐6 levels were quantified using enzyme‐linked immunosorbent assay (ELISA) and expressed in pg/mL. Correlation analysis shows the relationship between IL‐6 levels in HNEC‐ALI cultures exposed to exoproteins from planktonic (C) and biofilm (D) forms of *S. aureus* and *S. lugdunensis* during dual infections. Pearson's correlation coefficient was used for the analysis. HNEC‐ALI cultures were obtained from three independent donors (*n* = 3). The figure illustrates IL‐6 levels in response to planktonic (A) and biofilm (B) forms (Tukey's multiple comparisons test: *p* ≤ 0.05, **p ≤ 0.01, ***p ≤ 0.001; ns: not statistically significant).

We measured IL‐6 levels to evaluate the inflammatory response triggered following exposure of HNECs to *Staphylococcus* exoproteins. Exoproteins from *S. lugdunensis* in planktonic and biofilm form significantly induced IL‐6 production (−119.5 ± 42.14, *p* = 0.0165, and −145.3 ± 35.52, *p* = 0.0010 for planktonic and biofilm forms, respectively) from HNECs compared to control. However, no significant IL‐6 induction was seen when planktonic or biofilm forms of *S. aureus* or *S. epidermidis* exoproteins were applied to HNEC ALI cultures (Figure 5). IL‐6 production from HNECs exposed to various species were similar when isolates were co‐isolated with other species in dual or triple infections (Supporting Information S1: Figure S7).

During dual infection, HNECs exposed to exoproteins from *S. aureus* and *S. lugdunensis* correlated to elicit IL‐6 levels in both planktonic and biofilm forms (*r* = 0.7668, *p* = 0.0008, and *r* = 0.60, *p* = 0.0188, for planktonic and biofilm forms respectively) (Figure 5). However, no significant correlations were found in IL‐6 levels elicited by HNECs exposed to the exoproteins of *S. aureus* and *S. epidermidis* during dual infections in planktonic form (Supporting Information S1: Figure S8).

### 3.6 PCA Analysis Showing Species Distinct Impact on Nasal Epithelial Barrier Function, While the Severity of *S. aureus* Exoprotein‐Induced Nasal Barrier Disruption Correlates With the SNOT‐22 Severity Score

**FIGURE 6:**
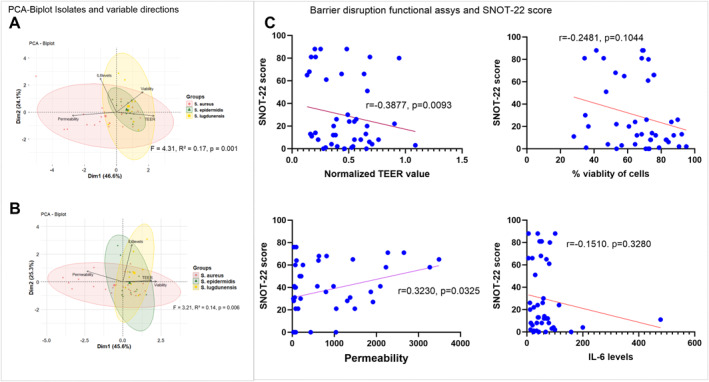
Principal component analysis (PCA) of TEER value, permeability, viability and IL‐6 levels following exposure to exoproteins of *S. aureus, S. epidermidis* and *S. lugdunensis* (A: planktonic, B: biofilm) on human nasal epithelial cell at air‒liquid interface (HNEC‐ALI) cultures. *S. aureus* exoprotein responses correlate with SNOT‐22 severity (C). Illustrating the correlation between TEER values, paracellular permeability, cell viability (%), and IL‐6 levels in HNEC‐ALI cultures exposed to exoproteins from various *S. aureus* strains and SNOT‐22 scores. Pearson's correlation coefficient was used for the analysis.

A Principal Component Analysis (PCA) was then performed on TEER, permeability, viability of cells, and IL‐6 levels of HNECs‐ALI cultures treated with 20 μg/mL of exoproteins of *S. aureus, S. epidermidis,* and *S. lugdunensis* isolates. Dim1 (46.6% variance) distinguished isolates with higher TEER and cell viability from those with elevated permeability, exhibiting barrier integrity distinctions. *S. aureus* isolates clustered toward the negative side of PC1, indicating strong barrier‐disruptive activity, while *S. epidermidis* localized near the origin, showing minimal epithelial impairment. *S. lugdunensis* isolates grouped on the positive side of Dim2 (24.1% variance), associated increase IL‐6 induction with minimal barrier disruption in planktonic form. A significant difference among *Staphylococcus* specie revealed through PERMANOVA analysis accounting 17% variance in barrier disruption parameters (*F* = 4.31, *R* ^2^ = 0.17, *p* = 0.001)]. A similar trend observed for biofilm forms (Figure 6A–B).

We next evaluated the relationship between CRS disease severity and measures of barrier disruption and inflammation induced by *Staphylococcus* exoproteins. *S. aureus* exoproteins showed a significant inverse correlation with TEER values (*r* = −0.3877, *p* = 0.0093) and a positive correlation with paracellular permeability (*r* = 0.323, *p* = 0.0325), while no significant correlations were observed for cell viability or IL‐6 production (Figure 6C). In contrast, no correlations were observed between exoproteins from *S. epidermidis* or *S. lugdunensis* and SNOT‐22 severity scores (Supporting Information S1: Figure S9).

### 3.7 Type 2 Immune Cell Infiltration Correlates With *S. aureus* Isolates That Induce IL‐6 Secretion Without Compromising Nasal Barrier Integrity

**FIGURE 7:**
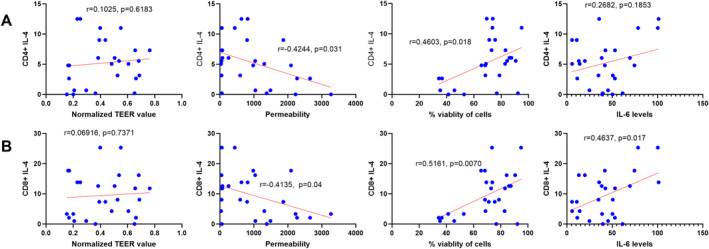
The correlation between immune cell markers (A) Th2 (CD4^+^ IL‐4^+^) and (B) Tc2 (CD8^+^ IL‐4^+^) cellular infiltration *and S aureus* exoprotein induced HNEC‐ALI culture functional and inflammatory assays. HNEC‐ALI culture functional and inflammatory assays included TEER values, paracellular permeability, cell viability (%), and IL‐6 levels. CD4^+^ IL‐4^+^ cells represent Type 2 Helper T cells (Th2 cells), while CD8^+^ IL‐4^+^ cells represent Type 2 Cytotoxic T cells (Tc2 cells). Pearson's correlation coefficient was used for the correlation analysis.

Finally, since severe CRS is characterized by type‐2 immune cell infiltration [37, 40], we investigated the relationship between *Staphylococcus* exoprotein‐induced barrier disruption and Type 2 T cell infiltration profiles investigated with flow cytometry on nasal polyps or sinus mucosa processed into single cell suspensions [29, 37, 40]. We observed inverse correlations between *S. aureus* exoprotein‐induced paracellular permeability and CD4^+^IL‐4^+^ Th2 (*r* = −0.4244, *p* = 0.031) and CD8^+^IL‐4^+^ Tc2 frequencies (*r* = −0.4135, *p* = 0.04). Positive correlations were found between cell viability and Th2 (*r* = 0.4603, *p* = 0.018) and Tc2 cells (*r* = 0.5161, *p* = 0.007). IL‐6 secretion positively correlated with Tc2 cell infiltration (*r* = 0.4637, *p* = 0.017) while similar but non‐significant trends were also observed with Th2 cell infiltration (Figure 7).

No significant correlations were observed between these immune markers and exoprotein‐induced effects on the barrier from *S. epidermidis* or *S. lugdunensis* exoproteins (Supporting Information S1: Figure S10).

## 4 Discussion

In this study, we investigated the impact of exoproteins from *S. aureus, S. epidermidis* and *S. lugdunensis,* isolated from the same sinonasal niche, on the nasal epithelial barrier. Our findings indicated that on average, exoproteins from *S. aureus* planktonic cells and biofilms caused significant, fast and sustained disruption of the nasal epithelial barrier, in contrast to the mild or negligible effect observed with exoproteins from *S. epidermidis* and *S. lugdunensis*. The severity of *S. lugdunensis* exoprotein‐induced barrier disruption, epithelial cytotoxicity and IL‐6 induction strongly correlated with the corresponding functional effects of co‐isolated *S. aureus* exoproteins. Correlation analysis showed that SNOT‐22 severity scores positively correlated with *S. aureus* exoprotein induced mucosal barrier disruption and IL‐6 production and inversely correlated with infiltration of type 2 inflammatory cells within nasal polyps and nasal mucosa. Together, these findings provide novel insights into the relationship between three commonly isolated *Staphylococcus* species in CRS and indicate strain‐dependent variation in their role in the disease process with *S. aureus* alone or in concert with *S. lugdunensis* as critical players in driving the disease.

Variations in the genetic makeup among *Staphylococci* encoding virulence factors, the capacity in biofilm formation and discrepancies in metabolic activities might account for the differences in the disruptive capabilities of these bacteria [41, 42, 43]. Numerous previous findings have indicated *S. aureus* to play a key role in the onset or perpetuation of CRS [44, 45, 46, 47, 48]. For example, it has been demonstrated that clinical *S. aureus* isolates triggered an immediate barrier disruption of HNEC‐ALI cultured cells through their secreted products in biofilm forms [47]. Similarly, *S. aureus* secreted extracellular proteases were found to impact HNEC‐ALI epithelial integrity in a dose and time dependent manner [48]. Hence, despite strain‐related differences, secreted products of *S. aureus* clinical isolates from the sinonasal cavity of CRS patients are widely recognized to impact the mucosal barrier [47, 49], in line with our findings. The application of exoproteins from *S. aureus* resulted in an immediate and sustained decline in TEER values, accompanied by decrease in cell viability, and elevated paracellular permeability indicating their disruptive effect on barrier function within the sinonasal niche. The disruptive impact of exoproteins from *S. aureus* can be credited to the pathogenic abilities of the bacteria, that express genes encoding various virulence factors though differing in efficiency [49, 50]. These include *α*‐toxin, super antigenic exotoxins, panton‐valentine leukocidin, and exfoliative toxin [21, 51]. These toxins have been found to facilitate membrane penetration, pore formation, and disruption of the physiological functions of nasal epithelial cells. Particularly, they damage tight junction proteins like claudins, occludin, and ZO‐1, compromising the permeability and enhancing bacterial invasion of the immune system and further tissue damage [21, 49, 52].

Compared to *S. aureus*, *S. epidermidis* and *S. lugdunensis*, on average, had less profound or no significant effects on disrupting epithelial barrier structure and function and were less toxic to cells with significant toxicity seen only after application of biofilm exoproteins. These findings are in line with the general consensus that those CoNS are less virulent than *S. aureus* and are often regarded as commensals. However, our findings indicate that not all CoNS strains are the same with the potential for certain *S. lugdunensis* strains, in particular the biofilm phenotypes, to have a pathogenic role. Namely, as for *S. aureus*, strain‐dependent variability was seen also for *S. epidermidis* and *S. lugdunensis* where certain strains could induce a decline in TEER values. The result aligns to a study conducted by Ramezanpour et al., where certain strains of *S. epidermidis* exoproteins cause a decline in the HNEC‐ALI cultured TEER values [23]. Interestingly, there were strong positive correlations between *S. aureus* and *S. lugdunensis* each time isolated from the same patient in their effect on TEER values, paracellular permeability, cell cytotoxicity and IL‐6 induction. This indicates both species might act in concert to affect the barrier and induce inflammation and supports the notion of host‐adaptation as seen to play a key role for survival of *S. aureus* over time despite the recurrent use of antibiotics [53]. In addition, there might be a sharing of genetic materials though HGT in between these species important to acquire new traits and adapt to changing environments contributing to the spread of antibiotic resistance genes, virulence factors, and other such traits [54]. This finding might also have therapeutic implications as targeting *S. aureus* only in a patient co‐infected with *S. lugdunensis*, for example by using *S. aureus* specific therapeutics like bacteriophages, might not be sufficient to eradicate infection and dampen inflammation. This hypothesis warrants further investigation.

IL‐6 is a pro‐inflammatory cytokine that plays a pivotal role in CRS pathophysiology. While physiological IL‐6 levels support immune regulation, tissue repair and remodeling [55], excessive production of IL‐6 and soluble IL‐6 receptor isoforms lead to dysregulated Th2‐mediated inflammation [56]. Thus, identifying bacterial contributions to IL‐6 release is critical for understanding CRS disease mechanisms. In our study, while variability was observed among individual isolates, *S. aureus* and *S. epidermidis* exoproteins did not induce a substantial increase in IL‐6 levels, consistent with previous reports [48]. This muted response may be attributed to epithelial cytotoxicity or the preferred induction of alternative cytokines. For example, *S. aureus‐*derived alpha‐toxin has been shown to stimulate IL‐5, IL‐10 and IL‐13 production [46], while *S. epidermidis* supernatants have been associated with increased IL‐8 and CXCL1 release [57]. In contrast, *S. lugdunensis* exoproteins significantly induced IL‐6 production without causing notable disruption to the epithelial barrier. This observation suggests a distinct immunomodulatory profile and underscores the need for further investigation into the specific components of *Staphylococcal* secreted products particularly those from *S. lugdunensis* that contribute to CRS pathogenesis.

The SNOT‐22 score is a validated, 22‐item questionnaire commonly used to assess symptom severity and quality of life in patients with CRS. It is widely used in both clinical and research settings to evaluate baseline severity and treatment outcomes [36]. In our study, logistic regression analysis revealed that *S. aureus* exoproteins from patients with higher SNOT‐22 scores induced significantly lower HNEC‐ALI cultured TEER values. Additionally, we observed a positive correlation between SNOT‐22 scores and paracellular permeability. These findings represent a novel observation that patients with more severe CRS symptoms tend to harbor *S. aureus* strains with greater capacity to disrupt the nasal epithelial barrier. Together, this not only reinforces the major role of *S. aureus* in CRS pathophysiology but also highlights exoprotein‐mediated barrier dysfunction as an objective marker of disease severity.

Our group previously reported that *S. aureus* biofilm properties and CRS severity scores were positively correlated with increased Th2 cell frequencies [40]. Th2 cells secrete cytokines such as IL‐4, IL‐5, and IL‐13, which are known to drive IgE production, eosinophilic inflammation, mucus hypersecretion, and tissue remodeling, all hallmarks of CRSwNP [58]. In the present study, we found that Th2 and Tc2 cell frequencies positively correlated with *S. aureus* exoproteins that caused minimal disruption to epithelial barrier integrity as indicated by low cell cytotoxicity and paracellular permeability. Notably, Th2/Tc2 cell infiltration also positively correlated with IL‐6 induction by *S. aureus* exoproteins. This was an unexpected finding, given that IL‐6 is known to suppress Th2 differentiation and promote Th17 differentiation. Although IL‐6 is enriched in CRSwNP tissues, it does not drive downstream STAT3 activation or IL‐17 production, suggesting an alteration in canonical IL‐6 signaling pathway [59]. Furthermore, IL‐6 has been reported to promote IL‐21 production in CD8^+^ T cells, which supports antibody responses within tertiary lymphoid structures [60]. Collectively, these findings suggest that *S. aureus* strains that do not overtly compromise epithelial integrity may still shape unique inflammatory profiles in CRS. The consequence of such immunomodulatory effects on CRS pathogenesis warrants further investigation.

One limitation of this study is the use of exoproteins rather than live bacteria, and other relevant nasal or respiratory bacterial species that are likely present in the sinonasal niche were not included. These species may interact in complex ways in vivo, which are currently difficult to replicate under in vitro conditions. Moreover, we have not fully explored all cytokines that may be induced in response to exposure of *Staphylococcus* exoproteins on HNEC ALI cultures. Thus, future studies are warranted incorporating a broader range of bacterial species and including in vivo models to better reflect the complexity of the nasal microbiota and their contribution to CRS.

In conclusion, this study provides novel insights into how *Staphylococcus* species differentially impact nasal epithelial barrier integrity, cytotoxicity, and inflammation. Strain‐specific virulence traits, particularly from *S. aureus* and *S. lugdunensis* isolated from the same niche, appeared to be coordinated and might act in synergy to contribute to mucosal damage and CRS pathogenesis. Notably, *S. lugdunensis* exoproteins induced stronger IL‐6 responses with limited cytotoxic or barrier‐disruptive effects while *S. aureus* exoproteins had stronger effects on disrupting the mucosal barrier. These findings highlight distinct mechanisms by which various *Staphylococcus* species may drive chronic inflammation in CRS.

## Author Contributions

The study was conceptualized and designed by S.A. under the supervision of S.V., K.A.F. and A.J.P., S.A. performed all experiments, analyzed and interpreted the data, and drafted the manuscript. M.R., C.M.C, G.S., I.B., E.F.B., P.J.W, S.V., K.A.F. and A.J.P. provided guidance on methodology, data analysis, interpretation of results, and manuscript revision. All authors reviewed and approved the final manuscript.

## Funding

This work was supported by the Australian National Health and Medical Research Council (NHMRC) [Grant 0006008606] to P.J.W. by the University of Adelaide Research Scholarship to S.A. and the Passe and Williams Foundation Senior Fellowship to S.V..

## Conflicts of Interest

The authors declare no conflicts of interest.

## Supporting information


Supporting Information S1

Table S1

## Data Availability

The datasets generated and/or analyzed for this study are available from the corresponding author upon reasonable request.
