# Supplementary material for: Staphylococcus aureus and Staphylococcus lugdunensis Act in Concert to Disrupt the Nasal Epithelial Barrier
Source: Clin Transl Allergy. 2026 May 22;16(5):e70177. doi: 10.1002/clt2.70177 (PMC13239409; doi:10.1002/clt2.70177)
Supplement: Supplementary file 1 — Supporting Information S1 [file CLT2-16-e70177-s001.docx]

**List of supplementary Figures**


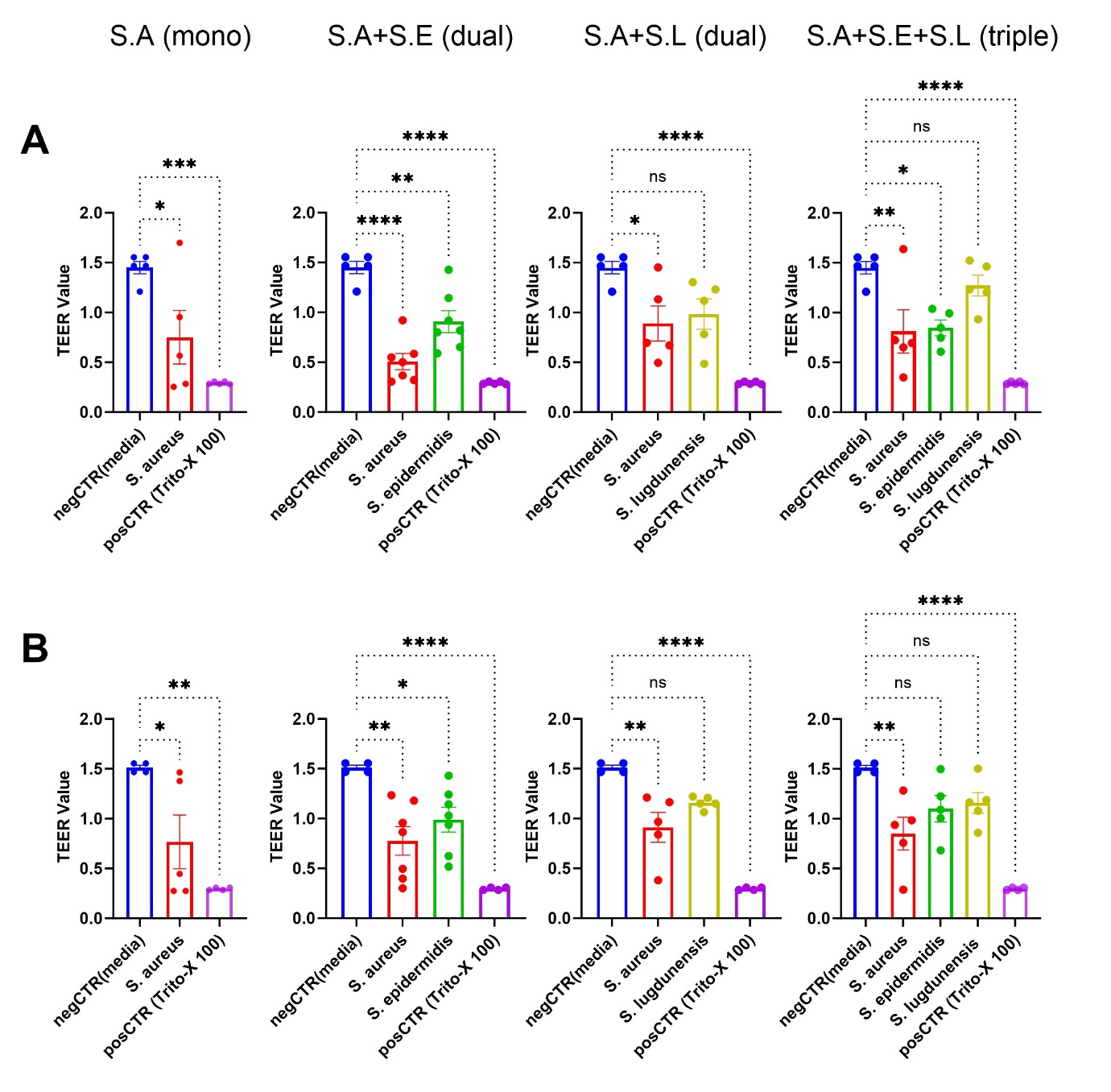


**Supplementary Figure 1: Transepithelial electrical resistance (TEER) of** **human nasal epithelial cell at air‒liquid interface (HNEC-ALI) cultures following exposure to exoproteins of *Staphylococcus* species during co-infections**. TEER value after exposure to exoproteins obtained from various *Staphylococci* species in planktonic (**A**) and biofilm (**B**) forms. *S. aureus* (S.A), *S. epidermidis* (S.E), and *S. lugdunensis* (S.L) either as mono- (S.A), dual- (S.A + S.E or S.A + S.L), or triple-species (S.A + S.E + S.L) infection. negCTR: untreated cells in Ex Plus medium; posCTR: cells treated with 1% Triton X-100. Data were analysed with the one-way ANOVA with Tukey's multiple comparisons test * p ≤0.05, ** p ≤0.01, ***p ≤ 0.001, **** p ≤ 0.0001; ns: not statistically significant.


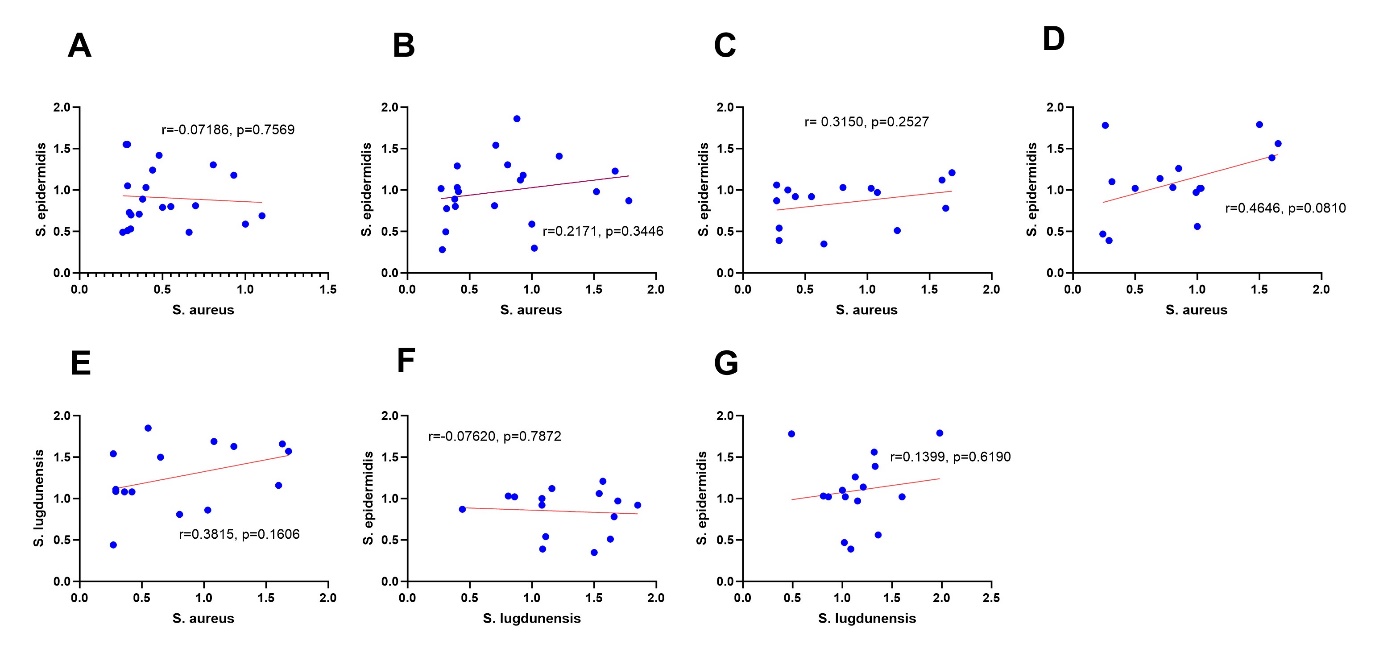


**Supplementary Figure 2: No correlation was observed in TEER values of HNEC-ALI cultures exposed to exoproteins of *S. aureus* and *S. epidermidis* isolated from same niche.** Correlation analysis of TEER values in HNEC-ALI cultures exposed to *S. aureus*, *S. epidermidis*, and *S. lugdunensis* in both planktonic (**A, C, E, F**) and biofilm (**B, D, G**) forms during dual (**A, B**) and triple (**C, D, E, F, and G**) infections. Pearson's correlation coefficient was used for the analysis.


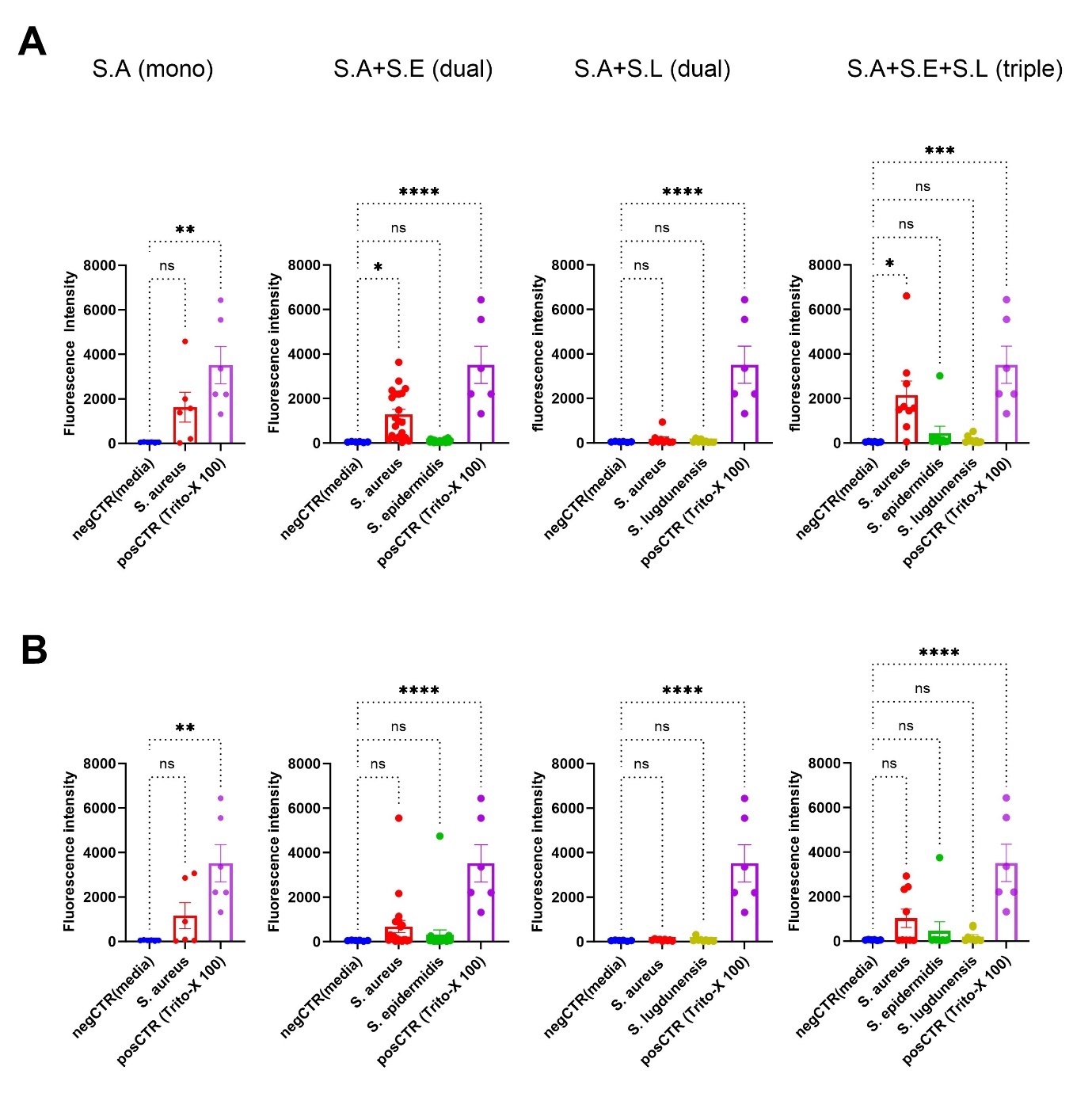


**Supplementary Figure 3:** **Paracellular permeability of human nasal epithelial cell at air‒liquid interface (HNEC-ALI) cultures following exposure to exoproteins of *Staphylococci* species during co-infections.** Paracellular permeability was measured by monitoring the passage of fluorescein isothiocyanate (FITC)-dextran across epithelial barrier. Paracellular permeability results after application of exoproteins obtained from various strains in planktonic forms (**A**) and biofilm forms (**B**). Data were analysed with the one-way ANOVA with Tukey's multiple comparisons test * p ≤0.05, ** p ≤0.01, ***p ≤ 0.001, **** p ≤ 0.0001; ns: not statistically significant. *S. aureus* (S.A), *S. epidermidis* (S.E), and *S. lugdunensis* (S.L) either as mono- (S.A), dual- (S.A + S.E or S.A + S.L), or triple-species (S.A + S.E + S.L) infection. negCTR: untreated cells in Ex Plus medium; posCTR: cells treated with 1% Triton X-100.


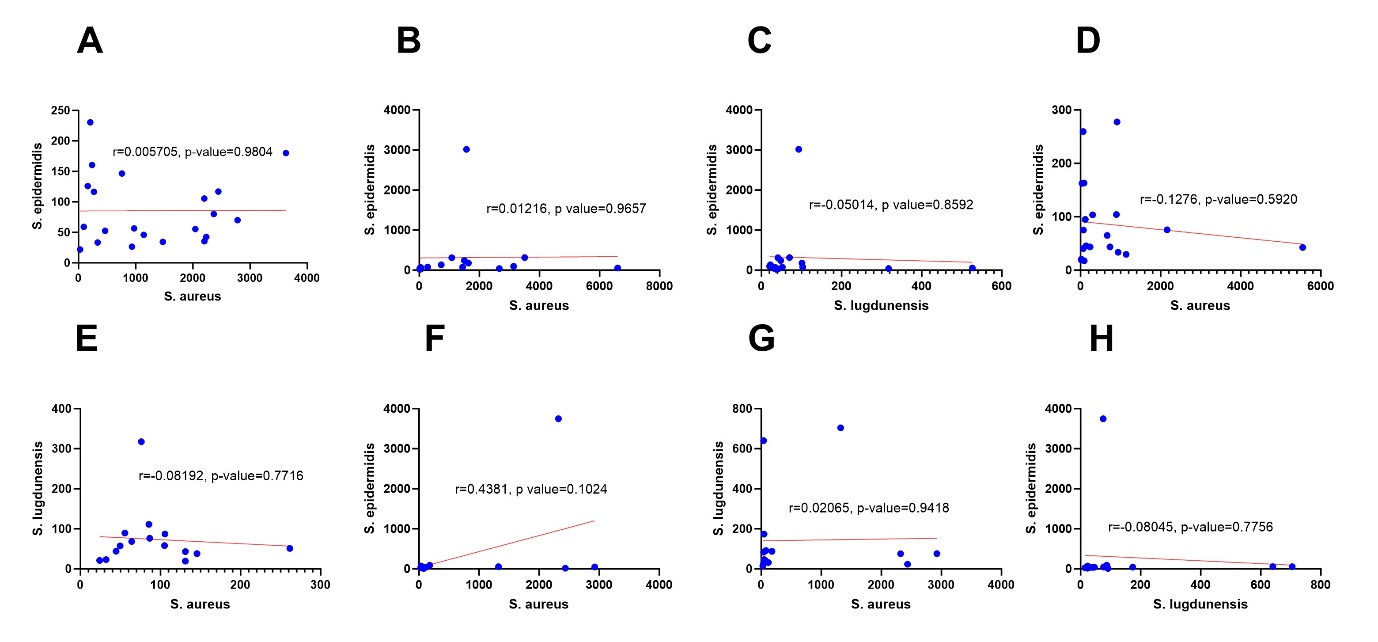


**Supplementary Figure 4**: The correlation analysis among the paracellular permeability of HNEC-ALI cultures exposed to *S. aureus,* *S. epidermidis,* and *S. lugdunensis* in both planktonic (**A, B,** and **C**) and biofilm (**D, E, F, G** and **H**) forms during dual (**A, D,** and **E**) and triple (**B, C, F, G** and **H**) infections. The Pearson rank correlation coefficient was performed for the correlation analysis.


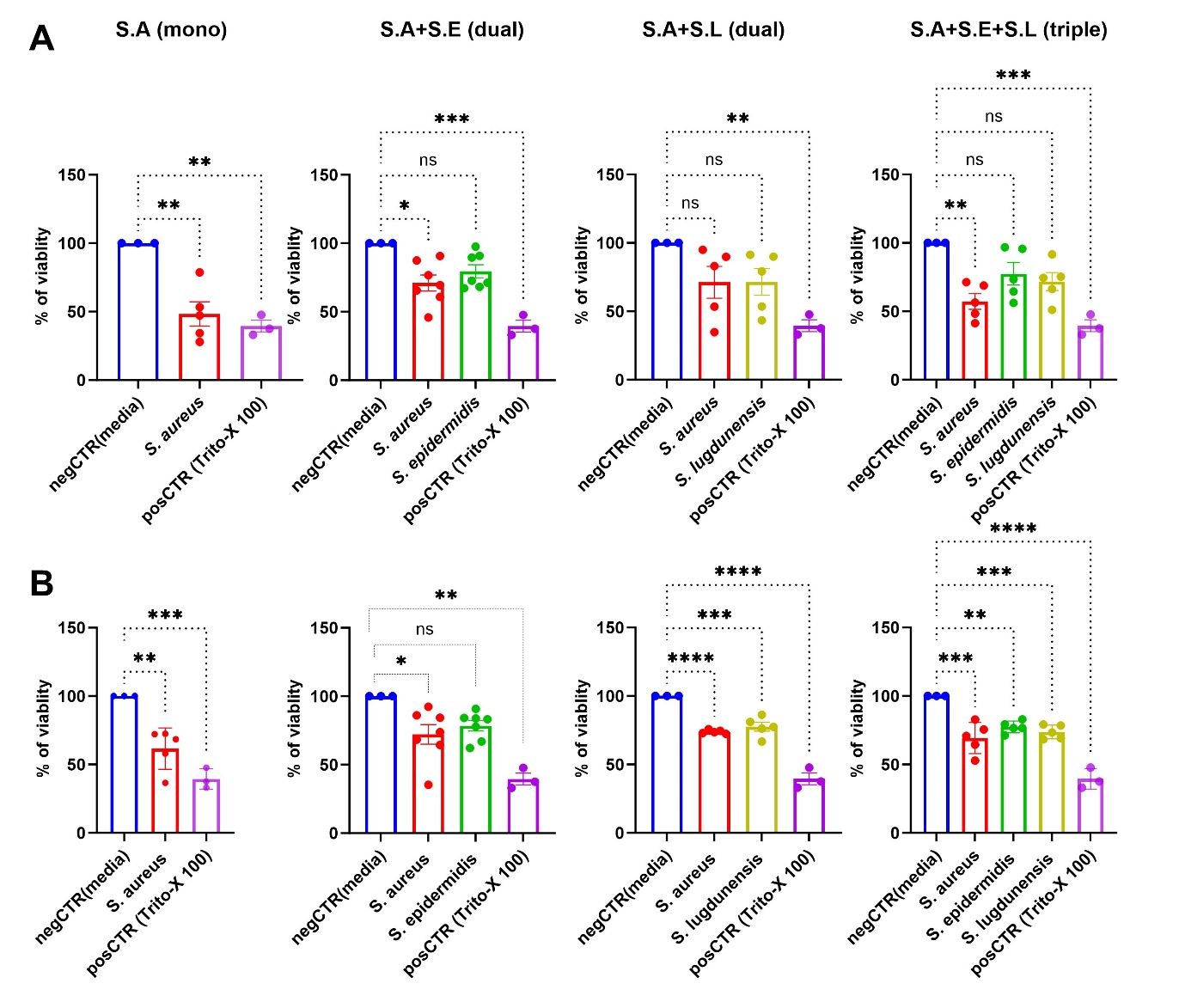


**Supplementary Figure 5:** **Cell** **Viability of human nasal epithelial cell at air‒liquid interface (HNEC-ALI) cultures following exposure to exoproteins of *Staphylococci* species during co-infections.** Cell viability was determined by lactate dehydrogenase (LDH) assay and the cell viability (%) was calculated relative to the negative control–treated cells. Planktonic forms (**A**) and biofilm forms (**B**) of viability results. Data were analysed with the one-way ANOVA with Tukey's multiple comparisons test * p ≤0.05, ** p ≤0.01, ***p ≤ 0.001, **** p ≤ 0.0001; ns: not statistically significant. *S. aureus* (S.A), *S. epidermidis* (S.E), and *S. lugdunensis* (S.L) either as mono- (S.A), dual- (S.A + S.E or S.A + S.L), or triple-species (S.A + S.E + S.L) infection. negCTR: untreated cells in Ex Plus medium; posCTR: cells treated with 1% Triton X-100.


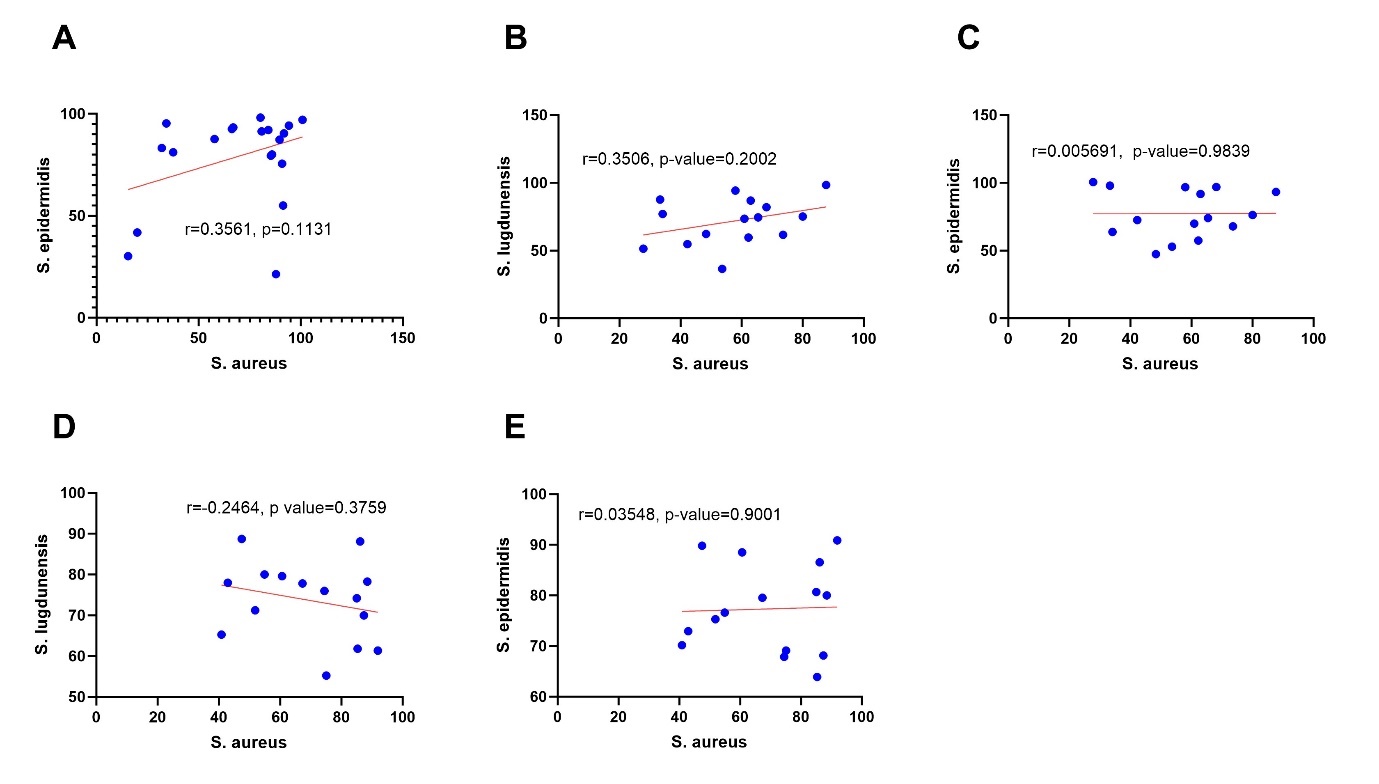


**Supplementary Figure 6:** The correlation describes the relationship between the percentage viability of cells exposed to exoproteins of planktonic (**A, B,** and **C**) and biofilm (**D, E**) forms of *S. aureus, S. epidermidis,* and *S. lugdunensis* isolated during dual (**A**) and triple (**B, C, D and E**) infections. The analysis was conducted using the Pearson rank correlation coefficient.


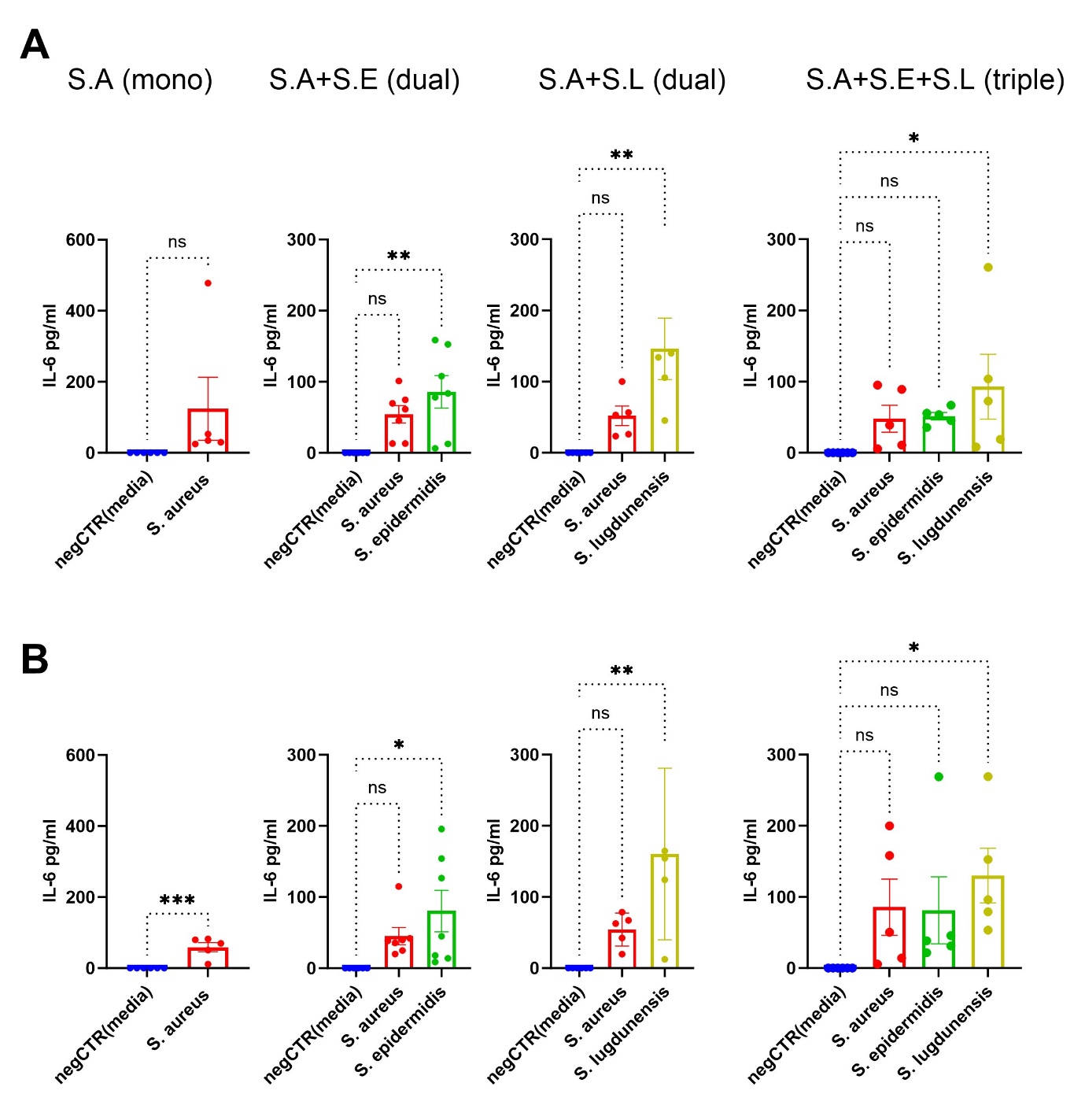


**Supplementary Figure 7: Interlukin-6 (IL-6)** **release in the human nasal epithelial cell at air‒liquid interface (HNEC-ALI) cultures following application of *Staphylococcus* species exoproteins during co-infections**. Enzyme-linked immunosorbent assay (ELISA) was used to quantify IL-6 concentrations (pg/mL), with results shown in planktonic (**A**) and biofilm (**B**) forms. Results analyzed using T-test and Tukey's multiple comparisons test *(** p ≤0.05, ** p ≤0.01, ***p ≤ 0.001; ns: not statistically significant). *S. aureus* (S.A), *S. epidermidis* (S.E), and *S. lugdunensis* (S.L) either as mono- (S.A), dual- (S.A + S.E or S.A + S.L), or triple-species (S.A + S.E + S.L) infection. negCTR: untreated cells in Ex Plus medium; posCTR: cells treated with 1% Triton X-100.
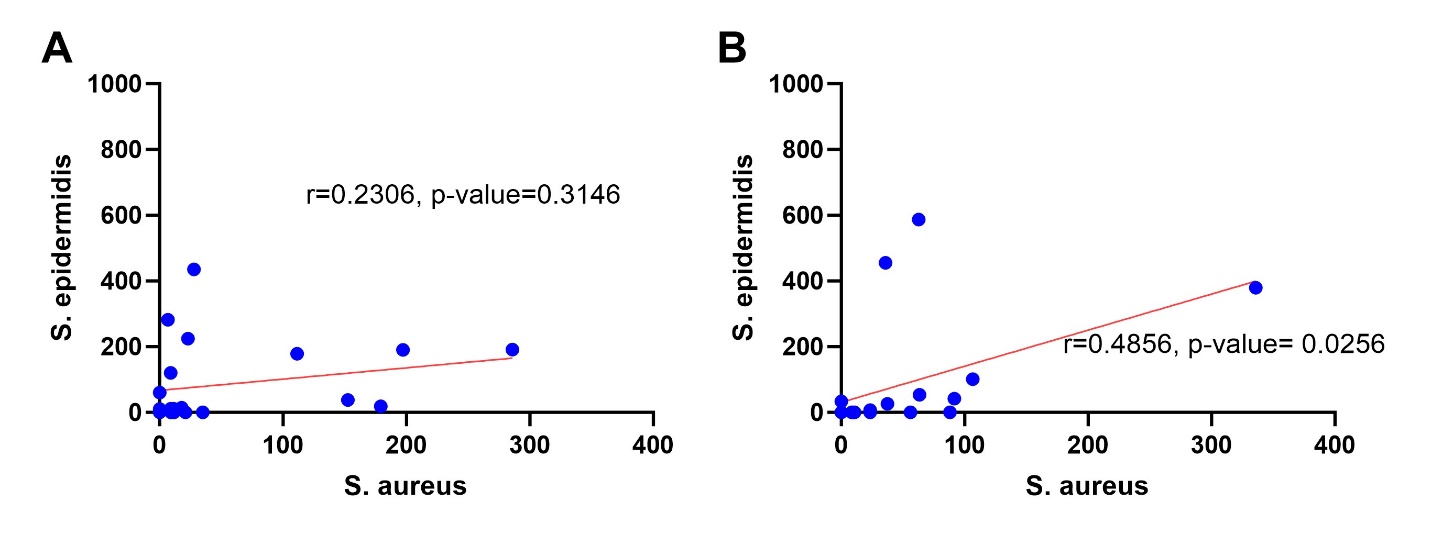


**Supplementary Figure 8:** **IL-6 release in HNEC-ALI cultures does not correlate following exposure to exoproteins from *S. aureus* and *S. epidermidis* during dual infections.** The correlation describes the relationship among IL-6 levels in HNEC-ALI cultures exposed to planktonic (**A**) and biofilm (**B**) forms of *S. aureus* and *S. epidermidis* during dual infections. Analysis was performed using the Pearson rank correlation coefficient.


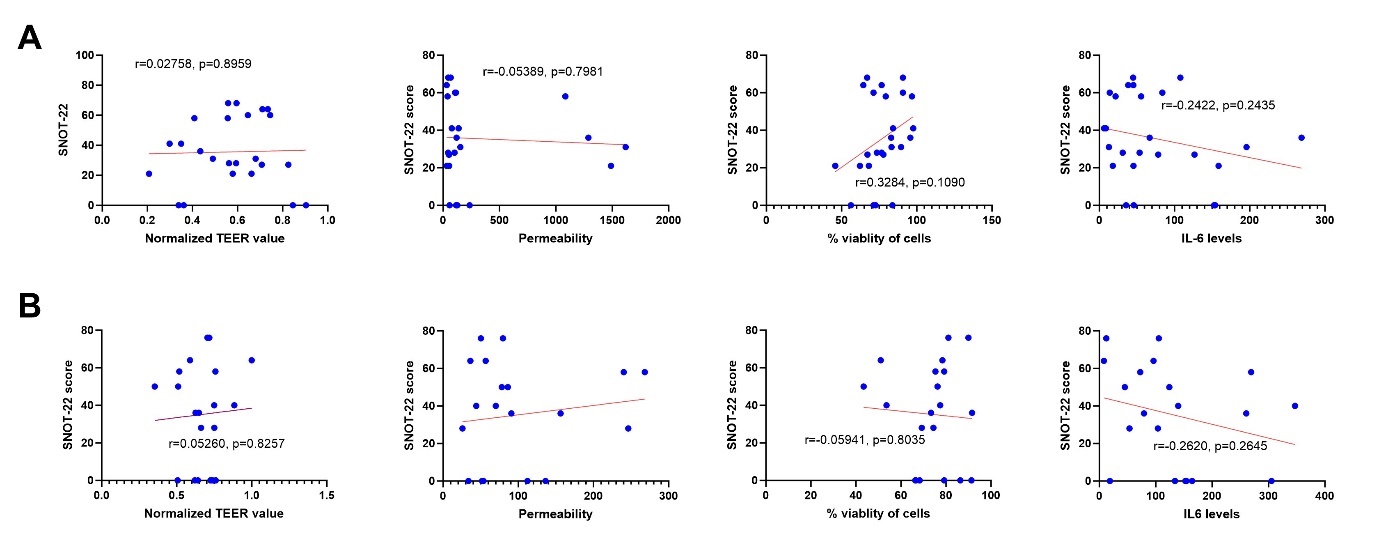


**Supplementary Figure 9: Human nasal epithelial cell at air‒liquid interface (HNEC-ALI) cultures functional and inflammatory assays does not correlate with SNOT-22 severity score in response to *S. epidermidis* and *S. lugdunensis* exoproteins** The figure describes the correlation analysis of the TEER value, paracellular permeability, % viability of cells and IL-6 levels of cells of HNEC-ALI cultures exposed to exoproteins of various strains of *S. epidermidis* (**A**) and *S. lugdunensis* (**B**) with SNOT-22*.* The Pearson rank correlation coefficient was used for the correlation analysis.


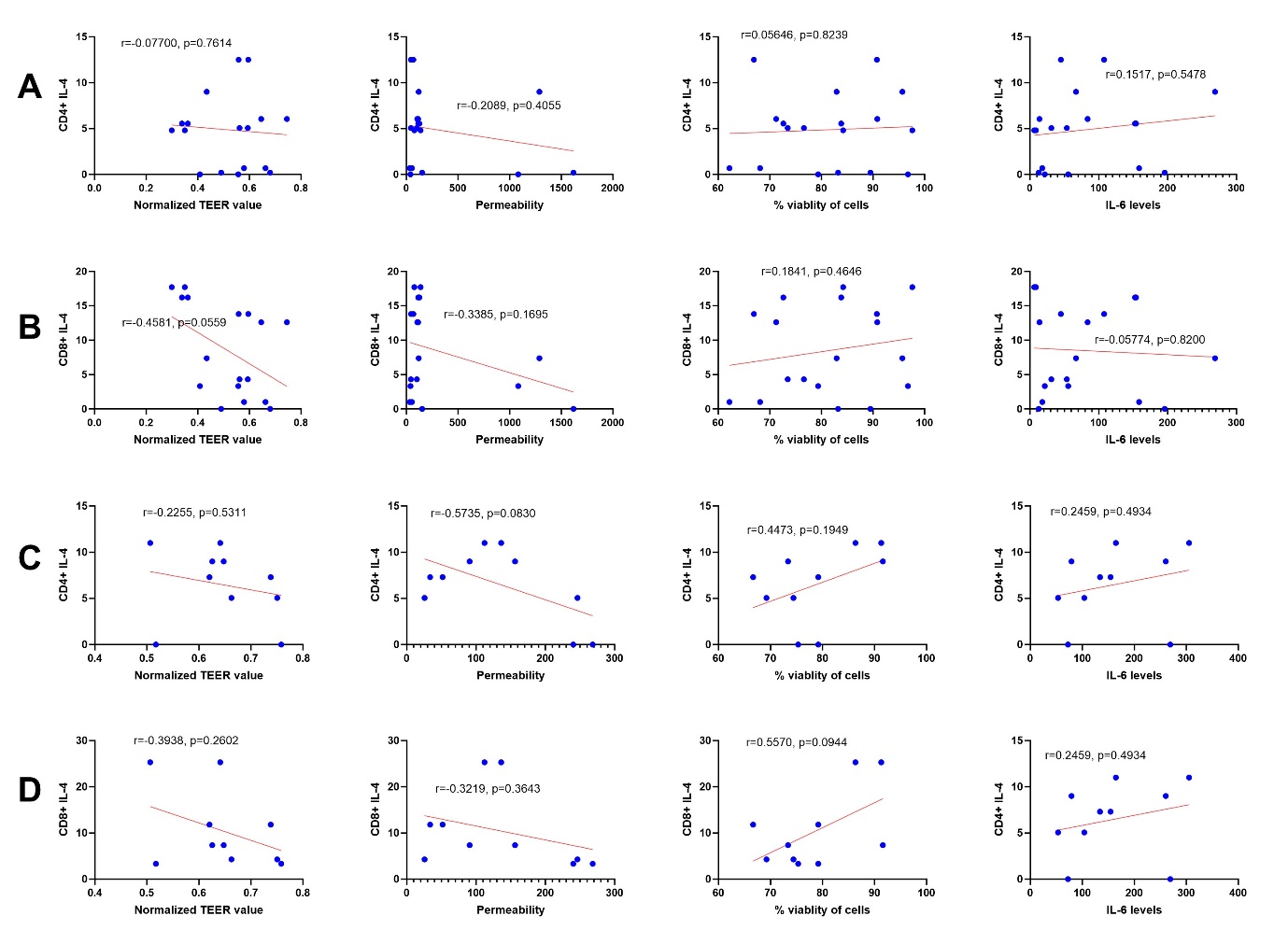


**Supplementary Figure 10: *S. epidermidis* and *S. lugdunensis* exoprotein-mediated effects on HNEC-ALI cultures do not correlate with Th2/Tc2 infiltration.** Correlation between (**A**) Th2 (CD4⁺ IL-4^+^) and (**B**) Tc2 (CD8⁺ IL-4^+^) cellular infiltration and *S. epidermidis* exoprotein-induced effects on HNEC-derived TEER, paracellular permeability, cell viability and IL -6 secretion*.* Correlation between (**C**) Th2 and (**D**) Tc2 cellular infiltration and *S.lugdunensis* HNEC-derived TEER, paracellular permeability, cell viability and IL -6 secretion*.* Correlation analyses were performed using Pearson’s correlation coefficient.
