# Supplementary material for: Staphylococcus aureus and Staphylococcus lugdunensis Act in Concert to Disrupt the Nasal Epithelial Barrier
Source: Clin Transl Allergy. 2026 May 22;16(5):e70177. doi: 10.1002/clt2.70177 (PMC13239409; doi:10.1002/clt2.70177)
Supplement: Supplementary file 2 — Table S1 [file CLT2-16-e70177-s002.docx]

**Supplementary Table 1**: Pairwise comparisons of *S. aureus* from different niches using Post Hoc Tukey’s test (planktonic and biofilm form)

| Measurement | Niche based *S. aureus* strains | | Planktonic form | | | | Biofilm form | | | |
| --- | --- | --- | --- | --- | --- | --- | --- | --- | --- | --- |
|  |  |  | MD | SE | t-value | p- value | MD | SE | t-value | p- value |
| Normalized TEER value | S.A(m) | S.A(e) | 0.161 | 0.145 | 1.112 | 0.687 | 0.106 | 0.138 | 0.772 | 0.866 |
|  |  | S.A(l) | -0.012 | 0.157 | -0.075 | 1.000 | 0.044 | 0.149 | 0.295 | 0.991 |
|  |  | S.A (t) | -0.015 | 0.157 | -0.094 | 1.000 | 0.072 | 0.149 | 0.487 | 0.961 |
|  | S.A(e) | S.A(l) | -0.173 | 0.145 | -1.194 | 0.638 | -0.062 | 0.138 | -0.453 | 0.968 |
|  |  | S.A (t) | -0.176 | 0.145 | -1.214 | 0.626 | -0.034 | 0.138 | -0.246 | 0.995 |
|  | S.A(l) | S.A (t) | -0.003 | 0.157 | -0.019 | 1.000 | 0.028 | 0.149 | 0.191 | 0.997 |
| Permeability | S.A(m) | S.A(e) | 119.286 | 593.908 | 0.201 | 0.997 | 105.414 | 449.503 | 0.235 | 0.995 |
|  |  | S.A(l) | 1237.900 | 641.494 | 1.930 | 0.251 | 692.9 | 485.519 | 1.427 | 0.5 |
|  |  | S.A (t) | -16.067 | 641.494 | -0.025 | 1.000 | 136.067 | 485.519 | 0.28 | 0.992 |
|  | S.A(e) | S.A(l) | 1118.614 | 593.908 | 1.883 | 0.270 | 587.486 | 449.503 | 1.307 | 0.57 |
|  |  | S.A (t) | -135.352 | 593.908 | -0.228 | 0.996 | 30.652 | 449.503 | 0.068 | 1 |
|  | S.A(l) | S.A (t) | -1253.967 | 641.494 | -1.955 | 0.241 | -556.833 | 485.519 | -1.147 | 0.666 |
| % viability of cells | S.A(m) | S.A(e) | -22.585 | 11.025 | -2.048 | 0.208 | -10.569 | 8.307 | -1.272 | 0.591 |
|  |  | S.A(l) | -22.913 | 11.908 | -1.924 | 0.253 | -12.308 | 8.973 | -1.372 | 0.532 |
|  |  | S.A (t) | -8.915 | 11.908 | -0.749 | 0.876 | -7.79 | 8.973 | -0.868 | 0.821 |
|  | S.A(e) | S.A(l) | -0.329 | 11.025 | -0.030 | 1.000 | -1.739 | 8.307 | -0.209 | 0.997 |
|  |  | S.A (t) | 13.670 | 11.025 | 1.240 | 0.611 | 2.779 | 8.307 | 0.335 | 0.987 |
|  | S.A(l) | S.A (t) | 13.999 | 11.908 | 1.176 | 0.649 | 4.518 | 8.973 | 0.503 | 0.957 |
| IL-6 levels | S.A(m) | S.A(e) | 69.945 | 57.459 | 1.217 | 0.624 | 13.782 | 28.535 | 0.483 | 0.962 |
|  |  | S.A(l) | 65.468 | 62.063 | 1.055 | 0.720 | 4.698 | 30.822 | 0.152 | 0.999 |
|  |  | S.A (t) | 76.131 | 62.063 | 1.227 | 0.619 | -26.858 | 30.822 | -0.871 | 0.819 |
|  | S.A(e) | S.A(l) | -4.477 | 57.459 | -0.078 | 1.000 | -9.084 | 28.535 | -0.318 | 0.988 |
|  |  | S.A (t) | 6.186 | 57.459 | 0.108 | 1.000 | -40.64 | 28.535 | -1.424 | 0.501 |
|  | S.A(l) | S.A (t) | 10.663 | 62.063 | 0.172 | 0.998 | -31.556 | 30.822 | -1.024 | 0.738 |

S.A(m): *S. aureus* during mono-infection, S.A(e): *S. aureus* during dual infection with *S. epidermidis*, S.A(l): *S. aureus* during dual infection with *S. lugdunensis*, S.A (t): *S. aureus* during triple infections. MD: mean difference, SE: standard error
